# Food Restriction Level and Reinforcement Schedule Differentially Influence Behavior during Acquisition and Devaluation Procedures in Mice

**DOI:** 10.1523/ENEURO.0063-23.2023

**Published:** 2023-09-26

**Authors:** Maxime Chevée, Courtney J. Kim, Nevin Crow, Emma G. Follman, Michael Z. Leonard, Erin S. Calipari

**Affiliations:** 1 Department of Pharmacology, Vanderbilt University, Nashville TN 37232; 2 Vanderbilt Brain Institute, Vanderbilt University, Nashville, TN 37232; 3 Vanderbilt Center for Addiction Research, Vanderbilt University, Nashville, TN 37232; 4 Department of Molecular Physiology and Biophysics, Vanderbilt University, Nashville, TN 37232; 5 Department of Psychiatry and Behavioral Sciences, Vanderbilt University, Nashville, TN 37232

**Keywords:** food restriction, goal directed, habits, operant conditioning, random intervals, random ratios

## Abstract

Behavioral strategies are often classified based on whether reinforcer value controls reinforcement. Value-sensitive behaviors, in which animals update their actions when reinforcer value is changed, are classified as goal-directed; conversely, value-insensitive actions, where behavior remains consistent when the reinforcer is removed or devalued, are considered habitual. Basic reinforcement schedules can help to bias behavior toward either process: random ratio (RR) schedules are thought to promote the formation of goal-directed behaviors while random intervals (RIs) promote habitual control. However, how the schedule-specific features of these tasks interact with other factors that influence learning to control behavior has not been well characterized. Using male and female mice, we asked how distinct food restriction levels, a strategy often used to increase task engagement, interact with RR and RI schedules to control performance during task acquisition and devaluation procedures. We determined that food restriction level has a stronger effect on the behavior of mice following RR schedules compared with RI schedules, and that it promotes a decrease in response rate during devaluation procedures that is best explained by the effects of extinction rather than devaluation. Surprisingly, food restriction accelerated the decrease in response rates observed following devaluation across sequential extinction sessions, but not within a single session. Our results support the idea that the relationships between schedules and behavioral control strategies are not clear-cut and suggest that an animal’s engagement in a task must be accounted for, together with the structure of reinforcement schedules, to appropriately interpret the cognitive underpinnings of behavior.

## Significance Statement

Understanding the basic learning principles that control behavior is essential to developing therapies for psychiatric disorders such as addiction or obsessive-compulsive disorder. Reinforcement schedules are thought to control the reliance on habitual versus goal-directed control during adaptive behaviors. However, external factors that are independent of training schedules also influence behavior, for example, by modulating motivation or energy balance. In this study, we find that food restriction levels are at least equally important as reinforcement schedules in shaping behavioral output. Our results add to a growing body of work which supports the argument for a nuanced distinction between habitual and goal-directed behavior.

## Introduction

Understanding how animals learn to control their actions is a fundamental goal of behavioral science. One useful framework classifies operant behaviors into two categories: those that are driven by the pursuit of a valuable goal and those that rely on value-insensitive processes (Graybiel, 2008; Smith and Graybiel, 2014). The assessment of these two types of behavior is done in operant conditioning tasks where behavior is maintained by the presentation of an appetitive reinforcer, typically sucrose or food. By devaluating the reinforcer before a probe test conducted under extinction conditions, either by providing *ad libitum* access to it or by pairing it with a negative consequence before the operant session, it is possible to determine whether a behavior is goal-directed: animals whose response rate drops following devaluation are considered to be implementing a goal-directed strategy while continued responding is evidence of a non goal-directed strategy (often referred to as habitual; Dickinson et al., 1983; Killcross and Coutureau, 2003; Gremel and Costa, 2013; Schreiner et al., 2020). Both the decreased value of the primary reinforcer and the degradation in action-outcome relationship experienced during the extinction sessions are thought to independently contribute to the decrease in response rate that reflects goal-directed control (Schreiner et al., 2020). Thus, this behavioral framework has guided a large amount of work focused on identifying the cognitive and neuronal processes distinguishing value sensitive and insensitive behaviors (Balleine and Dickinson, 1998; Yin and Knowlton, 2006; Graybiel and Grafton, 2015).

Two types of reinforcement schedules are known to engender different sensitivity to reinforcer devaluation, as assessed via extinction tests (Dickinson et al., 1983). In random ratio (RR) schedules, a reinforcer is delivered after a randomly selected number of responses is produced; this generates behavior that is goal-directed and sensitive to reinforcer devaluation procedures. Alternatively, random interval (RI) schedules allow access to a reinforcer after a randomly selected amount of time has elapsed. While interval schedules still require an operant response, responses are only reinforced following an elapsed period (Ferster and Skinner, 1957). These schedules often generate behavior over time that is insensitive to changes in the reinforcer and thus more resistant to extinction, when the reinforcer in omitted entirely (Dickinson et al., 1983). Although this dichotomy is a useful framework to study these types of behaviors, it has been challenged by experimental observations (Schreiner et al., 2020). For example, there is conflicting evidence regarding whether extended training on RR schedules becomes devaluation-insensitive over time (Adams and Dickinson, 1981; Adams, 1982; Dickinson, 1985; Miyachi et al., 1997) or remains goal-directed (Colwill and Rescorla, 1985, 1988; Colwill and Triola, 2002; Garr et al., 2021) and specific manipulations of the intervals used in RI schedules have been shown to produce behaviors that remain sensitive to reinforcer devaluation (Garr et al., 2020). Furthermore, the devaluation procedures used to evaluate whether animals are sensitive to the value of the reinforcer can suffer from the influence of extinction: during the probe extinction sessions that follow reinforcer/control devaluation, animals experience a degradation of the contingency between action and outcome that likely affects behavior (Delamater and Westbrook, 2014; Schreiner et al., 2020). Thus, while both devaluation and extinction affect value-dependent features of learning, the exact procedure implemented to perform devaluation tests may change the balance between the effects of devaluation and extinction on response rates.

While reinforcement schedule has a clear influence on operant behavior and sensitivity to devaluation procedures, schedule-independent factors, such as food restriction or stress, can also have effects on behavior that likely differ depending on the schedule under which the behavior occurs. For example, the same dose of systemic pentobarbital can increase or decrease the rate of food reinforcement depending on whether food delivery was reinforced under a ratio or interval schedule, respectively (Dews, 1955, 1956). In the behaviors discussed above, which are maintained by caloric foods, one particularly important factor is the food restriction state of the animal. Indeed, food intake is often restricted to increase response rates in operant tasks. While varying food restriction levels affects response rates and response patterns under certain conditions (Sidman and Stebbins, 1954; Powell, 1969; Lowe et al., 1974; Blakely and Schlinger, 1988; Schlinger et al., 1990), the effects of this common manipulation on RR compared with RI schedules, and on the subsequent sensitivity to devaluation procedures, are not known. Because RR schedules permit animals to control their own rate of reinforcement, while RI schedules necessarily constrain reinforcement rates to those allowed by the interval, we hypothesize that food restriction levels influence performance on RR schedules more strongly than performance on RI schedules and contribute to sensitivity to outcome devaluation procedures independently of schedule.

To explore this idea, we restricted the daily food intake of mice to various degrees and trained them on either RR or RI schedules for which we adjusted the number of responses required in RR schedules to match the actions-per-outcome mice of each restriction group achieved on an RI schedule. This strategy allowed us to mitigate the effect of differences in reinforcement efficiency and to compare the effects of food deprivation levels on performance during acquisition and devaluation procedures across RI and RR schedules. We found that deprivation had a combinatorial effect on operant behavior: increased food restriction was more effective at increasing response rates under RR compared with RI schedules. Furthermore, devaluation tests showed that the order of session, rather than the type of prefeeding, drove the decrease in response rates we observed during extinction tests. This decrease was different across food restriction groups such that more restricted animals extinguished their responses more strongly than mildly restricted animals but was unaffected by the training schedule. Surprisingly, this difference only manifested across consecutive extinction sessions but was not apparent within a single session.

Together, our results demonstrate that external factors such as food restriction level must indeed be accounted for, together with the structure of reinforcement schedules, to appropriately interpret the cognitive underpinnings of behavior.

## Materials and Methods

### Subjects

Experiments were approved by the Institutional Animal Care and Use Committee of Vanderbilt University Medical Center and conducted according to the National Institutes of Health *Guidelines for Animal Care and Use*. Eighty-seven eight-week-old animals were used for this study. C57BL/6J mice (43 males and 44 females) were acquired from The Jackson Laboratory (SN: 000664) and maintained on an 8 A.M./8 P.M. 12/12 h reverse light/dark cycle. Experiments were performed during the dark phase. Animals were single housed for the duration of the study with unlimited access to water.

The experiments were performed in five separate rounds using different mice each time.

1. Twenty mice (10 females, 10 males) were mildly food restricted (3 g/d) for one week. They were then trained on FR1 until acquisition criteria were met (see Procedure below) and assigned to a RR10/RR20 schedule or RI30/RI60 schedule such that time to acquisition and sex were balanced across the two groups. This training session resulted in *N* = 5 females, 5 males performing an RR10/RR20 task under “Mild Restriction” conditions and *N* = 4 females, 5 males performing an RI30/RI60 task under “Mild Restriction” conditions (one female failed to acquire FR1 for 10 consecutive days).
2. Twenty mice (10 females, 10 males) were mildly food restricted (3 g/d) for one week. They were then trained on FR1 until acquisition criteria were met (see Procedure below) and assigned to either a “No Restriction” group, which had unlimited access to food, or to a “Strong Restriction” group which received 2 g chow per day, such that time to acquisition and sex were balanced across the two groups. All animals went on to perform an RI30/RI60 task. This training session resulted in *N* = 5 females, 5 males performing an RI30/RI60 task under “No Restriction” conditions and *N* = 5 females, 5 males performing an RI30/RI60 task under “Strong Restriction” conditions.
3. Twenty mice (10 females, 10 males) were mildly food restricted (3 g/d) for one week. They were then trained on FR1 until acquisition criteria were met (see Procedure below) and assigned to either a “No Restriction” group, which had unlimited access to food, or to a “Strong Restriction” group which received 2 g chow per day, such that time to acquisition and sex were balanced across the two groups. Mice assigned to the “No Restriction” group then followed an RR2.8/RR3.4 schedule (ratios matched to the performance of the “No Restriction” group on RI30/RI60) and mice assigned to the “Strong Restriction” group followed an RR5.1/RR9.3 schedule (ratios matched to the performance of the “Strong Restriction” group on RI30/RI60). This training session resulted in *N* = 4 females, 5 males performing an RR2.8/RR3.4 task under “No Restriction” conditions (one female failed to acquire FR1 for 10 consecutive days) and *N* = 3 females, 5 males performing an RR5.1/RR9.3 task under “Strong Restriction” conditions (one female was a statistical outlier and one female was erroneously given unlimited access to food in the middle of the experiment and removed from the cohort).
4. Ten mice (five females, five males) were mildly food restricted (3 g/d) for one week. They were then trained on FR1 until acquisition criteria were met (see Procedure below) and then followed a RR3.4/RR5.2 schedule (ratios matched to the performance of the “Mild Restriction” group on RI30/RI60). This training session resulted in *N* = 5 females, 5 males performing an RR3.4/RR5.2 task under “Mild Restriction” conditions.
5. Seventeen mice (eight males, nine females) were trained to increase sample sizes of all groups and perform additional control experiments (postdevaluation preference tests). Mice were mildly food restricted (3 g/d) for one week and trained on FR1 until acquisition criteria were met. They were then split such that time to acquisition and sex were balanced across groups as follows: 5 mice (3 males, 2 females) were mildly food restricted (3 g/d) and trained on RI30/RI60; 4 mice (1 male, 3 females) were strongly food restricted (2 g/d) and trained on RI30/RI60; 5 mice (2 males, 3 females) were mildly food restricted (3 g/d) and trained on RR3.4/RR5.2; 2 mice (1 male, 1 female) were strongly food restricted (2 g/d) and trained on RR5.1/RR9.3. One mouse did not acquire FR1 within 10 d. This additional cohort underwent postdevaluation preference tests to confirm the efficacy of the devaluation procedure.

The number of animals included in each analysis is specified in the figure legends.

### Apparatus

Mice were trained and tested daily in individual standard-wide mouse operant conditioning chambers (Med Associates Inc.) outfitted with a retractable lever. A 3D-printed divider was inserted in each chamber, limiting the available space to a small square area providing access to a sucrose port and a lever (area: 13 × 13 = 169 cm^2^). A custom-made 3D-printed wall insert was used to hold and display a stainless-steel cannula (lick port, 18 gauge, 0.042′′ ID, 0.05′′ OD, 0.004′′ wall thickness), which was connected to a syringe pump for sucrose delivery. An illumination light was affixed above the lick port.

### Procedure

#### General procedural information

All sessions lasted until the maximum number of rewards was obtained (51) or 1 h was reached, whichever came first. White noise signaled the beginning of the session and was on for the entire duration of the session. All animals but a subset of the RI_MildRestriction group (*N* = 9) were weighed before each session.

#### Task design

Mice were first trained on a fixed ratio 1 (FR1) schedule of reinforcement. Each lever press resulted in the delivery of 8 μl of a 10% sucrose solution. Additional presses performed after sucrose delivery but before sucrose collection had no programmed consequence and did not count toward obtaining the next reinforcer. Once the reinforcer was collected, lever presses counted again. Mice were considered to have met the acquisition criteria once they had obtained 51 rewards within the allotted 1 h for 2 consecutive days. Upon meeting the criteria, mice were assigned to different tasks (see above, Subjects) such that time to acquisition and sex were balanced across groups.

##### Random interval (RI) training

RI schedules consisted of 3 d operating under an RI30 schedule, in which a lever press resulted in sucrose delivery only after an interval of time averaging 30 s (range: 0–125 s, generated according to $f\left(x\right)=(\frac{1}{\mu })e^{-\frac{x}{\mu }},$ where μ = 30) had passed since consuming the last reinforcer, followed by 4 d operating under an RI60 schedule (range: 0–208 s).These intervals were determined in keeping with previous studies, which suggested that this training procedure produces habitual actions (Gremel and Costa, 2013; Hilário et al., 2007).

##### Random ratio (RR) training

**Figure 1.**
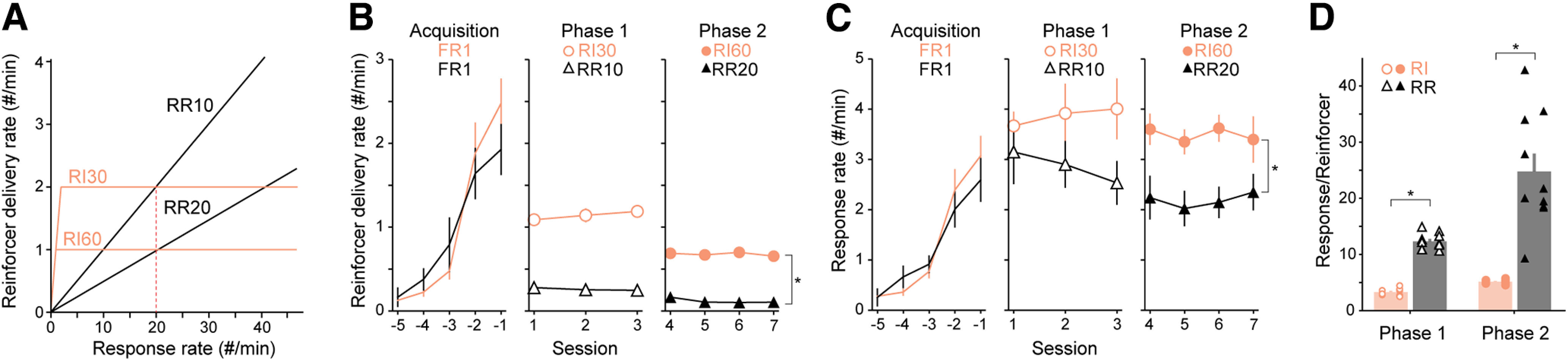
RI30/RI60 and RR10/RR20 produce different operant behavior and different response-per-reinforcer ratios. ***A***, Schematic showing the relationship between response rate and reinforcer delivery rate under four example schedules. The red dotted line indicates the mean response rate a subject must perform to achieve equal reinforcer delivery rates under RI30 or RR10 schedules. The value is the same for achieving equal reinforcer delivery rates between RI60 and RR20. ***B***, Mean reinforcer delivery rates across training, split by schedule. ***C***, Mean response rates across training, split by schedule. ***D***, Mean responses-per-reinforcer ratio during each phase of training. (Note: animals on RR20 can have mean response/reinforcer ratio larger than 20 because responses only counted once the previous reinforcer had been collected.) Individual data points are shown separated by sex within each group (Females: left, Males: right). RI: *N* = 9 mice, RR: *N* = 10 mice, * indicates independent Student’s *t* test *p* < 0.05. Data are shown as mean ± SEM (error bars are occluded by mean symbol in panel ***B***). See also Extended Data Figure 1-1.

**Figure 2.**
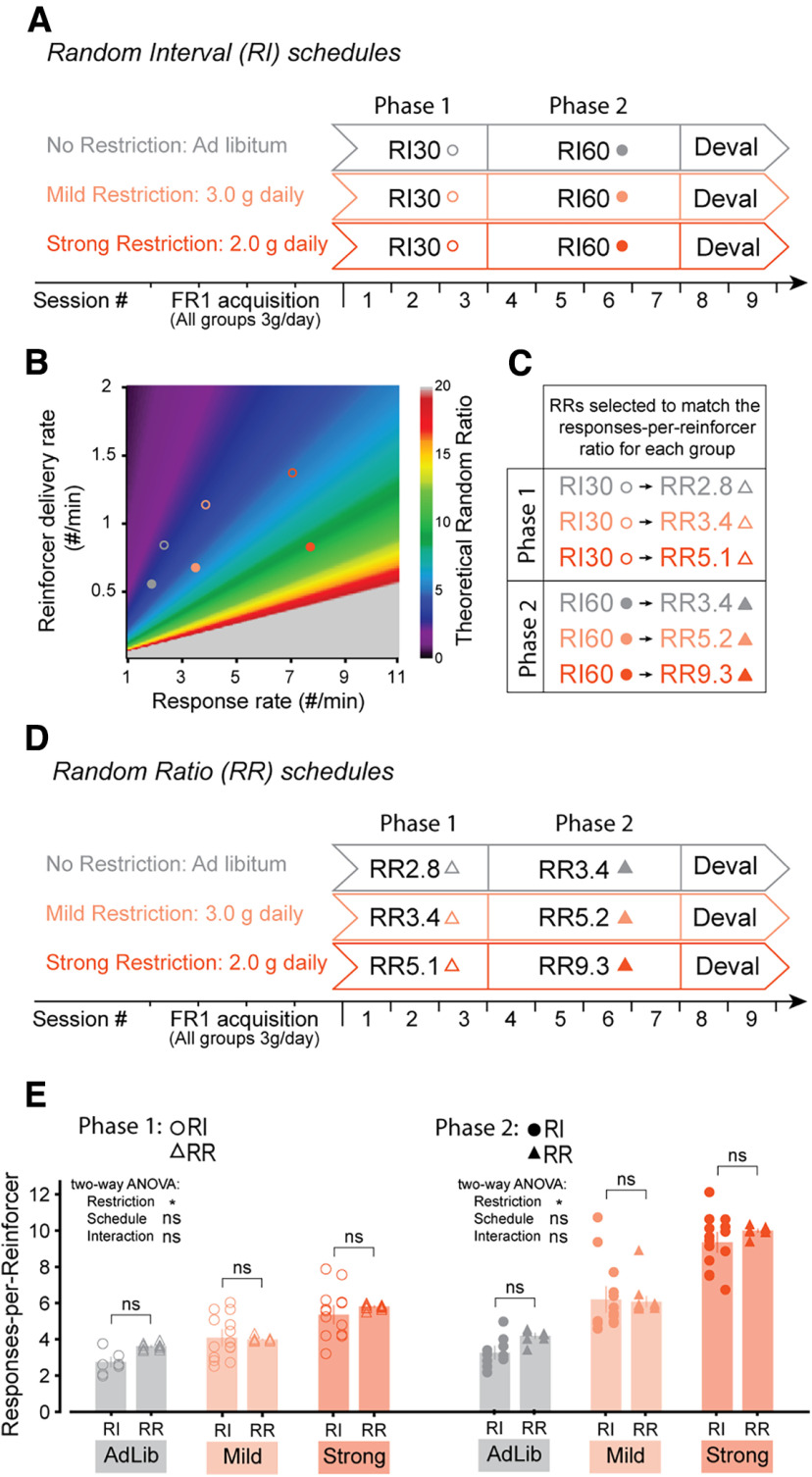
Designing RR schedules to match the responses-per-reinforcer ratios observed in mice following RI schedules under distinct levels of food restriction. ***A***, Schematic showing the training strategy for three groups distinguished by their level of food restriction. Each mouse underwent 3 d of RI30, 4 d of RI60, and 2 d of outcome devaluation testing. ***B***, Plot showing that each pair of response rate/reinforcer delivery rate can be achieved by a specific RR (shown as a color map). The response rate/reinforcer delivery rate values observed for each phase/restriction group are depicted as circles color-coded to match ***A***. ***C***, Summary of ***B*** showing the RR that matches each RI based on the performance of each RI group. ***D***, Schematic showing the training strategy for three groups distinguished by their level of food restriction. Each group followed the RR schedule identified in ***B***, ***C***. ***E***, Mean responses-per-reinforcer ratios for each restriction/schedule group, separated by phase of training. Individual data points are shown separated by sex within each group (Females: left, Males: right). RI-noRestriction *N* = 10 mice, RR-noRestriction *N* = 9 mice, RI-MildRestriction *N* = 14 mice, RR-MildRestriction *N* = 15 mice, RI-strongRestriction *N* = 14 mice, RR-strongRestriction *N* = 10 mice; * indicates two-way ANOVA *p* < 0.05 (***E***). ns indicates two-way ANOVA *p* > 0.05 (***E***) and *post hoc* Tukey’s HSD test *p* > 0.5 (***E***). Data are shown as mean ± SEM. See also Extended Data Figure 2-1.

For the cohort reported in Figure 1, the RR schedules consisted of 3 d operating under an RR10 schedule, in which a lever press resulted in sucrose delivery only after a specific amount of responses (10 on average, range: 0–44, generated according to $f\left(x\right)=(\frac{1}{\mu })e^{-\frac{x}{\mu }},$ where μ = 10) had been performed, followed by 4 d operating under an RR20 schedule (20 on average, range: 0–73). For the three cohorts with distinct food restriction levels, the ratios were calculated to match the responses-per-reinforcer ratio of their counterparts who performed the RI task and are detailed in Result (Fig. 2*B*,*C*) as well as in Subject above. No-Restriction RR2.8 (range: 0–11), RR3.4 (range: 0–14); Mild-Restriction RR3.4 (range: 0–14), RR5.1 (range: 0–19); Strong-Restriction RR5.2 (range: 0–21), RR9.3 (range: 0–45).

10.1523/ENEURO.0063-23.2023.f1-1 Extended Data Figure 1-1 Statistical table. Download Figure 1-1, XLSX file.

10.1523/ENEURO.0063-23.2023.f2-1 Extended Data Figure 2-1 No difference in time to acquisition across restriction/schedule and in the efficacy of food restriction across sex. ***A***, Graph showing the time to acquisition across training schedules and restriction groups [RI-NoRestriction: 4.1 ± 0.5 sessions (*N* = 10), RI-MildRestriction: 4.29 ± 0.62 sessions (*N* = 14), RI-StrongRestriction: 3.86 ± 0.49 sessions (*N* = 14), RR-NoRestriction: 3.78 ± 0.52 sessions (*N* = 9), RR-MildRestriction: 3.93 ± 0.45 sessions (*N* = 15), RR-StrongRestriction: 3.4 ± 0.45 sessions (*N* = 10)]. A two-way ANOVA revealed no effects of restriction group (two-way ANOVA *F* = 0.45, df = 2, *p* = 0.64) and no effect of task schedule (two-way ANOVA *F* = 0.77, df = 1, *p* = 0.38). These results are expected as we assigned mice to groups post FR1 acquisition to avoid any differences. ***B***, Graph showing the effects of food restriction across time as the fraction of initial body weight [data on session 7: Females/NoRestriction: 1.02 ± 0.02 (*N* = 9), Females/MildRestriction: 0.99 ± 0.03 (*N* = 10), Females/StrongRestriction: 0.79 ± 0.01 (*N* = 12), Males/NoRestriction: 1.02 ± 0.01 (*N* = 10), Males/MildRestriction: 0.92 ± 0.02 (*N* = 10), Males/StrongRestriction: 0.76 ± 0.01 (*N* = 12)]. A two-way ANOVA on the last day (session 7) revealed a significant effect of restriction group (two-way ANOVA *F* = 90.1, df = 2, *p* = 2.3e-18) but no effect of sex (two-way ANOVA *F* = 3.48, df = 1, *p* = 6.7e-2). Download Figure 2-1, TIF file.

#### Outcome devaluation tests

Upon completing the 7 d of RI or RR training, each mouse underwent an outcome devaluation test. This procedure consists of providing access to the reinforcer (10% sucrose, devalued condition) or to another familiar food source that was not used as a reinforcer (chow, valued condition) *ad libitum* for 1 h before a 10-min extinction session, in which the lever presses have no programmed consequences and do not result in reinforcer delivery. Within each cohort, half the mice were assigned to perform this test “Day1: valued, Day2: devalued” or “Day1: devalued, Day2: valued.” Mice which consumed <0.2 × *g* during either prefeeding sessions were excluded from the analysis of outcome devaluation tests, as well as one RR-StrongRestriction animal whose response rate during the extinction session was a statistical outlier (z = 3.6), resulting in a dataset of *N* = 41 mice included in these analyses (RI-MildRestrcition *N* = 8, RI-StrongRestriction *N* = 14, RR-MildRestriction *N* = 10, RR-StrongRestriction *N* = 9). A devaluation index was calculated for each mouse $\frac{R_{\mathrm{valued}}}{R_{\mathrm{valued}}+R_{\mathrm{devalued}}},$ where *R_valued_* is the response rate during the valued extinction session and *R_devalued_* is the response rate during the devalued extinction session), such that an index close to 1 indicates sensitivity to outcome devaluation (i.e., goal-directed control). Similarly, the extinction index was defined as $\frac{R_{2nd}}{R_{1st}+R_{2nd}},$ where *R_1st_* is the response rate during the first extinction session and *R_2nd_* is the response rate during the second extinction session) for across sessions and as $\frac{R_{2nd}}{R_{1st}+R_{2nd}},$ where *R_1st_* is the response rate during the first minute of the first extinction session and *R_2nd_* is the response rate during the second minute of the first extinction session) for within session analyses.

#### Postdevaluation preference test

A subset of mice (*N* = 16, fifth cohort described in Subjects above) underwent an additional test to validate the effectiveness of the devaluation procedure. Following each extinction session, mice were given free access to both sucrose and chow for 10 min. The amounts consumed were measured and a preference index was used to determine whether mice preferred to consume what they had not had access to during the devaluation period preceding the extinction test. The preference index following sucrose devaluation was defined as:

$$\frac{S_{S}}{S_{S}+S_{C}}-\frac{C_{S}}{C_{S}+C_{C}},$$

S_S_ = sucrose consumed during preference test following sucrose devaluation.

S_C_ = sucrose consumed during preference test following chow devaluation.

C_S_ = chow consumed during preference test following sucrose devaluation.

C_C_ = chow consumed during preference test following chow devaluation.

Thus, an index close to 1 indicates preference for sucrose and an index close to −1 indicates preference for chow.

### Analysis

#### Statistical analyses

**Figure 3.**
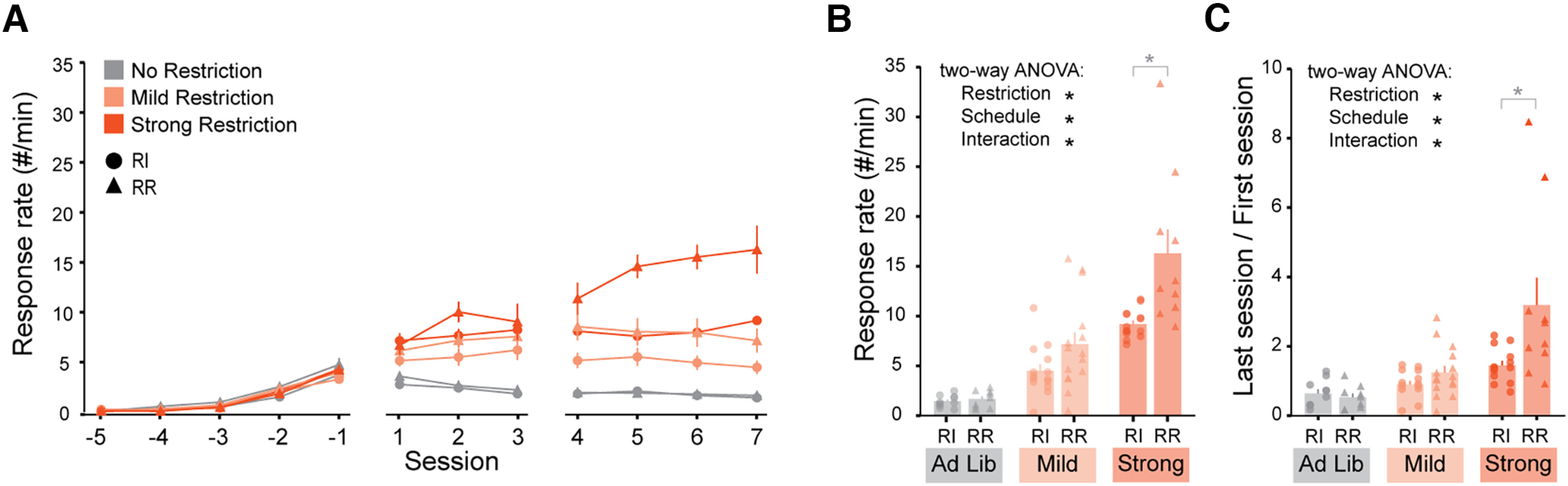
Food restriction increases response rates more effectively in mice following RR schedules than in mice following RI schedules. ***A***, Mean response rates across training, split by restriction and schedule. ***B***, Mean response rates on the last day of training summarizing the data used to perform a two-way ANOVA. ***C***, Mean ratio of response rate on last session over first session (after FR1 acquisition) summarizing the data used to perform a two-way ANOVA. Individual data points are shown separated by sex within each group (Females: left, Males: right). RI-noRestriction *N* = 10 mice, RR-NoRestriction *N* = 9 mice, RI-MildRestriction *N* = 14 mice, RR-MildRestriction *N* = 15 mice, RI-strongRestriction *N* = 14 mice, RR-strongRestriction *N* = 10 mice; black * indicates two-way ANOVA *p* < 0.05 (***B***, ***C***); gray * indicates *p* < 0.05 *post hoc* Tukey’s HSD test for matched RR-RI pairs (***B***, ***C***). ANOVA results and *post hoc* Tukey’s HSD tests are reported in the statistical table (Extended Data Fig. 1-1). Data are shown as mean ± SEM. See also Extended Data Figure 3-1.

**Figure 4.**
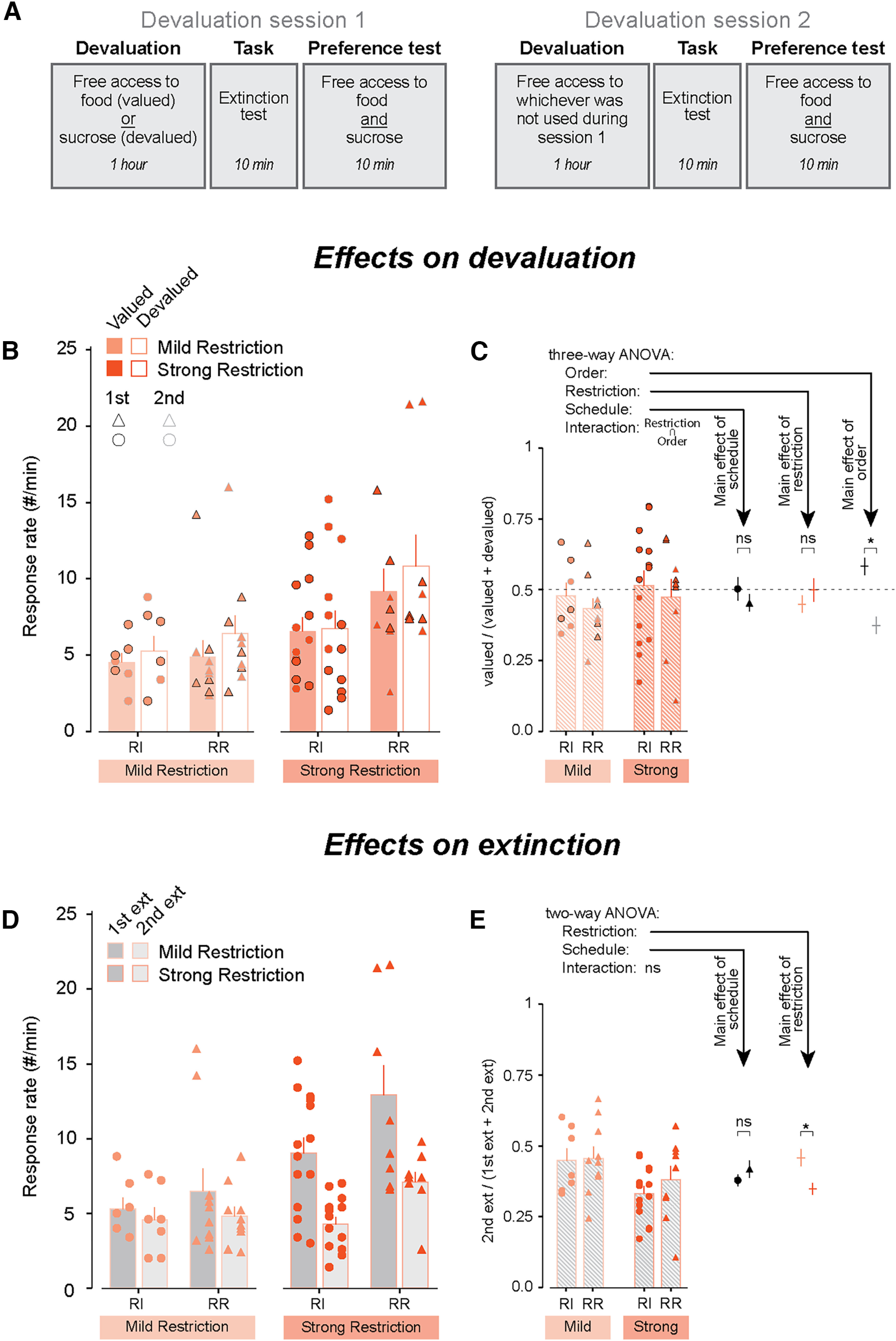
The effects of food restriction and schedule on performance during devaluation procedures are dominated by extinction. ***A***, Diagram describing the devaluation procedure, extinction test, and preference test. ***B***, Summary of response rates during devaluation sessions, grouped by devaluation (valued/food, devalued/sucrose). Valued conditions consisted of 1-h access to regular chow and devalued 1-h access to 10% sucrose. The two test sessions were performed on consecutive days and the order counterbalanced within each group (valued-RI-MildRestriction: 4.3 ± 0.56 responses/min, devalued-RI-MildRestriction: 4.95 ± 0.91 responses/min, *N* = 8; valued-RR-MildRestriction: 4.88 ± 1.08 responses/min; devalued-RR-MildRestriction: 6.40 ± 1.21 responses/min, *N* = 10; valued-RI-StrongRestriction: 6.54 ± 0.92 responses/min; devalued-RI-StrongRestriction: 6.74 ± 1.16 responses/min, *N* = 14; valued-RR-StrongRestriction: 9.18 ± 1.46 responses/min; devalued-RR-StrongRestriction: 10.82 ± 2.05 responses/min, *N* = 9). ***C***, Same data as shown in ***B*** summarized as a devaluation index for each mouse [valued response rate/(valued response rate + devalued response rate)]. Means for the mice grouped by schedule, restriction and testing order are also shown on the right. ***D***, Same data as shown in ***B***, grouped by session number (first extinction session, second extinction session; first-RI-MildRestriction: 4.95 ± 0.74 responses/min, second-RI-MildRestriction: 4.30 ± 0.77 responses/min, *N* = 8; first-RR-MildRestriction: 6.48 ± 1.08 responses/min; second-RR-MildRestriction: 4.80 ± 0.62 responses/min, *N* = 10; first-RI-StrongRestriction: 9.01 ± 1.05 responses/min; second-RI-StrongRestriction: 4.27 ± 0.47 responses/min, *N* = 14; first-RR-StrongRestriction: 12.91 ± 1.99 responses/min; second-RR-StrongRestriction: 7.09 ± 0.66 responses/min, *N* = 9). ***E***, Same data as shown in ***D*** summarized as an extinction index for each mouse [second session response rate/(first session response rate + second session response rate)]. Means for the mice grouped by schedule and by restriction are also shown on the right. * indicates two-way ANOVA *p* < 0.05 (***C***, ***E***). ns indicates two-way ANOVA *p* > 0.05 (***C***, ***E***). Data are shown as mean ± SEM. Individual data points are shown separated by sex within each group (Females: left, Males: right). See also Extended Data Figure 4-1.

**Figure 5.**
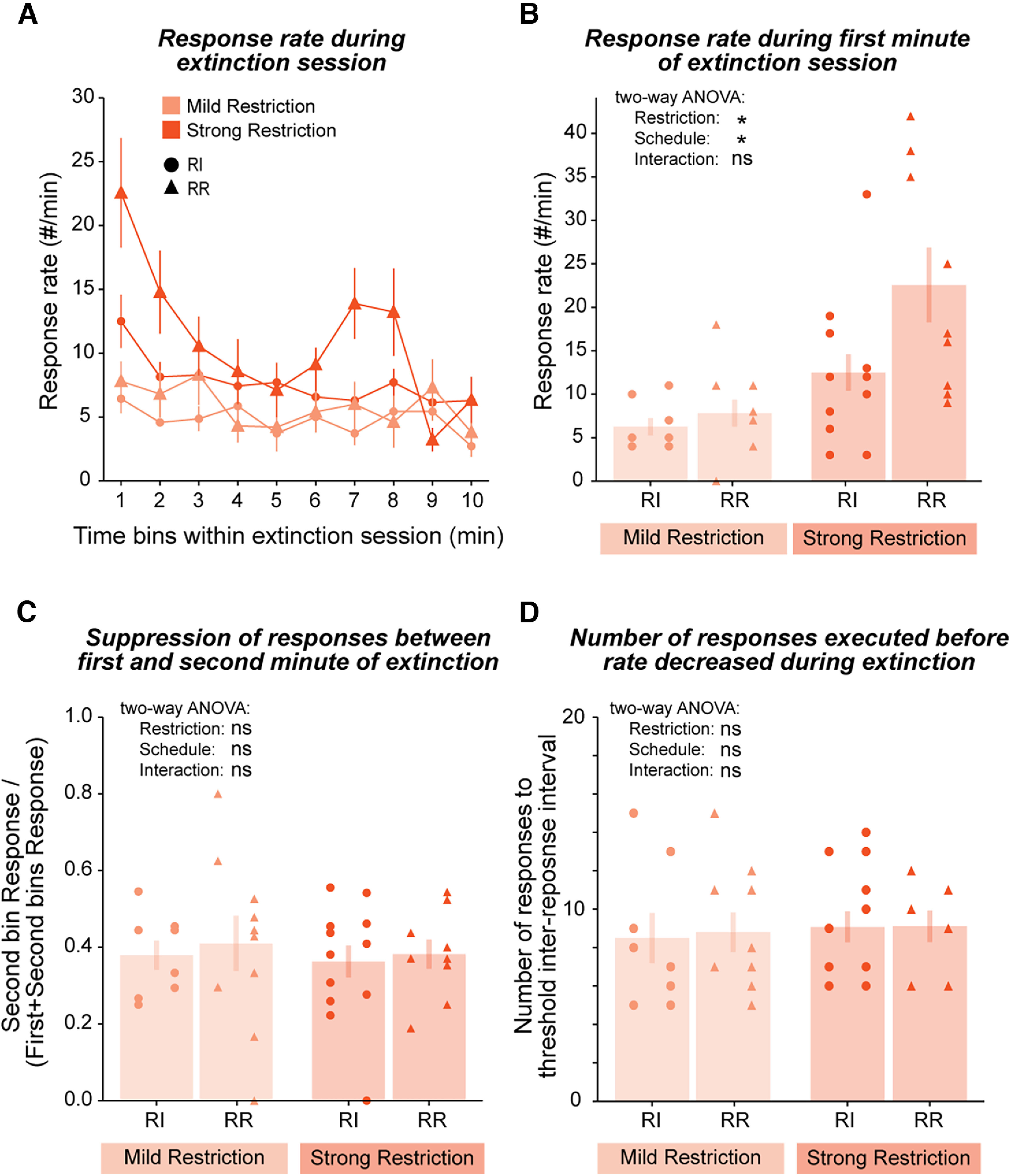
The effects of food restriction on extinction are not apparent within a session. ***A***, Response rates during the first extinction session. Data are shown across the duration of the session in 1 min time bins. ***B***, Response rates during the first minute of the extinction session show similar results as by the end of acquisition. ***C***, Graph showing the ratio of the number of responses in the second minute over the number of responses during the first and second minutes capturing the magnitude of the decrease in response rate. ***D***, Graph showing the number of responses executed before the inter-response interval became larger than mean ± 2 SD based on a rolling window of five responses. * indicates two-way ANOVA *p* < 0.05 (***B***). ns indicates two-way ANOVA *p* > 0.05 (***B–D***). Data are shown as mean ± SEM. Individual data points are shown separated by sex within each group (Females: left, Males: right). RI-MildRestriction: *N* = 8, RR-MildRestriction: *N* = 10, RI-StrongRestriction: *N* = 14, RR-StrongRestriction: *N* = 9).

All analyses were performed using custom code in Python (v3.6.13). The SciPy package (v1.5.3) was used to perform independent Student’s *t* tests (Fig. 1*B–D*) and the Pingouin package (v0.3.12) was used to perform two-way and three-way ANOVAs as well as the corresponding *post hoc* Tukey’s tests (Figs. 2*E*, 3*B*,*C*, 4*C*,*E*, 5*B–D*; Extended Data Figs. 2-1*A*,*B*, 3-1*B*,*D*, 4-1*A–C*). All data are reported as mean ± SEM and all statistical tests used are specified in Results or in figure legends as well as in the statistical table (Extended Data Fig. 1-1).

10.1523/ENEURO.0063-23.2023.f3-1 Extended Data Figure 3-1 Food restriction and schedule influenced reinforcer delivery rates and response rate variability. ***A***, Mean reinforcer delivery rates across training, split by restriction and schedule. ***B***, Mean reinforcer delivery rates on the last day of training summarizing the data used to perform a two-way ANOVA. ***C***, Mean coefficient of variation of the inter-response interval across training, split by restriction and schedule. ***D***, Mean coefficient of variation on the last day of training summarizing data used to perform a two-way ANOVA. RI-noRestriction *N* = 10 mice, RR-NoRestriction *N* = 9 mice, RI-MildRestriction *N* = 14 mice, RR-MildRestriction *N* = 15 mice, RI-strongRestriction *N* = 14 mice, RR-strongRestriction *N* = 10 mice; black * indicates two-way ANOVA *p* < 0.05 (***B***, ***D***); grey * indicates *p* < 0.05 *post hoc* Tukey’s HSD test for matched RR-RI pairs (***B***, ***D***). ANOVA results and *post hoc* Tukey’s HSD tests are reported in the statistical table. Data are shown as mean ± SEM. Individual data points are shown separated by sex within each group (Females: left, Males: right). Download Figure 3-1, TIF file.

10.1523/ENEURO.0063-23.2023.f4-1 Extended Data Figure 4-1 Prefeeding selectively and effectively reduced mice’s preference for the prefed food. ***A***, Graphs showing how much food [left; RI-MildRestriction: 1.08 ± 0.24 g (*N* = 8), RR-MildRestriction: 0.94 ± 0.17 g (*N* = 10), RI-StrongRestriction: 1.25 ± 0.09 g (*N* = 14), RR-StrongRestriction: 1.52 ± 0.16 g (*N* = 10); two-way ANOVA main effect of restriction group df = 1, *F* = 4.35, *p* = 0.044; main effect of schedule df = 1, *F* = 0.15, *p* = 0.70; interaction df = 1, *F* = 2.39, *p* = 0.13) or sucrose (right; RI-MildRestriction: 1.62 ± 0.44 g (*N* = 8), RR-MildRestriction: 1.65 ± 0.40 g (*N* = 10), RI-StrongRestriction: 2.9 ± 0.22 g (*N* = 14), RR-StrongRestriction: 3.16 ± 0.18 g (*N* = 10); two-way ANOVA main effect of restriction group df = 1, *F* = 18.6, *p* = 1.15e-4; main effect of schedule df = 1, *F* = 0.24, *p* = 063; interaction df = 1, *F* = 0.15, *p* = 0.70] mice consumed during the 1 h prefeeding session preceding the extinction test. ***B***, Graphs showing consumption during the postdevaluation preference test, during which mice have free access to both food and sucrose for 10 min, for food [left; RI-MildRestriction_postSucDeval: 0.52 ± 0.04 g (*N* = 5), RI-MildRestriction_postFoodDeval: 0.24 ± 0.07 g (*N* = 5), RR-MildRestriction_postSucDeval: 0.48 ± 0.09 g (*N* = 4), RR-MildRestriction_postFoodDeval: 0.27 ± 0.11 g (*N* = 4), RI-StrongRestriction_postSucDeval: 0.45 ± 0.05 g (*N* = 4), RI-StrongRestriction_postFoodDeval: 0.15 ± 0.05 g (*N* = 4), RR-StrongRestriction_postSucDeval: 0.55 ± 0.05 g (*N* = 2), RR-StrongRestriction_postFoodDeval: 0.10 ± 0.10 g (*N* = 2); three-way ANOVA main effect of restriction group df = 1, *F* = 1.68, *p* = 0.21; main effect of schedule df = 1, *F* = 0.014, *p* = 0.91; main effect of prefeed df = 1, *F* = 30.8, *p* = 1.4e-5; interactions all showed *p* > 0.05) and for sucrose (right; RI-MildRestriction_postSucDeval: 0.24 ± 0.11 g (*N* = 5), RI-MildRestriction_postFoodDeval: 1.26 ± 0.28 g (*N* = 5), RR-MildRestriction_postSucDeval: 0.22 ± 0.05 g (*N* = 4), RR-MildRestriction_postFoodDeval: 1.22 ± 0.36 g (*N* = 4), RI-StrongRestriction_postSucDeval: 0.10 ± 0.04 g (*N* = 4), RI-StrongRestriction_postFoodDeval: 1.35 ± 0.28 g (*N* = 4), RR-StrongRestriction_postSucDeval: 0.15 ± 0.15 g (*N* = 2), RR-StrongRestriction_postFoodDeval: 0.65 ± 0.55 g (*N* = 2); three-way ANOVA main effect of restriction group df = 1, *F* = 0.59, *p* = 0.45; main effect of schedule df = 1, *F* = 0.59, *p* = 0.45; main effect of prefeed df = 1, *F* = 33.5, *p* = 8.0e-6; interactions all showed *p* > 0.05]. ***C***, Preference index based on data shown in ***B*** showing all groups preferred the nondevalued option equally. Negative values indicate preference for food and positive values indicate preference for sucrose [following sucrose devaluation, RI-MildRestriction: −0.59 ± 0.12 (*N* = 5), RR-MildRestriction: −0.45 ± 0.15 (*N* = 4), RI-StrongRestriction: −0.66 ± 0.05 (*N* = 4), RR-StrongRestriction: −0.76 ± 0.24 (*N* = 2); indices following food devaluation are the opposite, two-way ANOVA main effect of restriction group df = 1, *F* = 1.49, *p* = 0.25; main effect of schedule df = 1, *F* = 0.15, *p* = 0.70; interaction df = 1, *F* = 0.74, *p* = 0.41]. Data are shown as mean ± SEM. Individual data points are shown separated by sex within each group (Females: left, Males: right). Download Figure 4-1, TIF file.

## Results

### RI and RR schedules in mice can produce different response rates and different responses-per-reinforcer ratios

The stronger relationship between action and outcome in RR schedules compared with RI schedules is often cited as the primary reason for differences in sensitivity to outcome devaluation (Dickinson, 1985; Pérez et al., 2019; although see Garr et al., 2020). Mice on RR can increase their reinforcer rate by increasing their response rate while the rate of reinforcer delivery on RI schedules does not correlate with response rate (Fig. 1*A*). Therefore, an animal’s response rate can result in distinct responses-per-reinforcer ratios that are dependent on schedule. To illustrate this phenomenon, we compared food-restricted mice trained on RI and RR schedules using commonly used ratios/intervals (Hilário et al., 2007; Gremel and Costa, 2013). First, we restricted the mice’s daily food intake to 3 g/d to increase their engagement in the task and trained them to respond for sucrose under a fixed ratio 1 (FR1) schedule of reinforcement. Upon acquisition of FR1, mice went on to a two-phase schedule comprised of either (1) 3 d of RI30 followed by 4 d of RI60 or (2) 3 d of RR10 followed by 4 d of RR20. When reinforcer delivery rate (Fig. 1*B*, Extended Data Fig. 1-1 RI: 0.67 ± 0.02 reinforcers/minute, *N* = 9; RR: 0.12 ± 0.02 reinforcers/minute, *N* = 10) and response rate were assessed (Fig. 1*C*, RI: 3.43 ± 0.13 responses/minute, *N* = 9; RR: 2.19 ± 0.30 responses/minute, *N* = 10), we found that during Phase 2 the RI group had both a faster reinforcer delivery rate (independent Student’s *t* test *p* = 5.3e-14) and a faster response rate (independent Student’s *t* test *p* = 0.0021) compared with the RR group. This result indicates that, under these food restriction conditions, an RI30/RI60 training schedule is more effective than a RR10/RR20 schedule at increasing reinforcer and response rates.

Interestingly, the response-per-reinforcer ratio was much larger for the RR group than for the RI group during both phases of training (Fig. 1*D*, Phase 1, RI: 3.33 ± 0.21, *N* = 9; RR: 12.3 ± 0.45, *N* = 10, Welch’s *t* test *p* = 2.2e-10; Phase 2, RI: 5.11 ± 0.12, *N* = 9; RR: 24.8 ± 3.19, *N* = 10, Welch’s *t* test *p* = 1.6e-4). In other words, reinforcer rates between RR and RI schedules were not comparable (they would only have been comparable at 20 responses/min; Fig. 1*A*, dotted line). Thus, the differences in behavior following training under RI versus RR tasks are confounded with differences in response rates, which themselves are determined by reinforcement rate. We next set out to test whether the reinforcement rate or the schedule contingency better predicted operant performance and sensitivity to devaluation procedures.

### Designing RR schedules to match the responses-per-reinforcer ratios observed in mice following RI30/RI60 schedules under distinct levels of food restriction

To determine whether differences in response rates rather than differences in schedule contingencies best explain operant performance and sensitivity to reinforcer devaluation procedures, we first had to tailor RR schedules to produce a similar relationship between response rate and reinforcer delivery (responses-per-reinforcer) compared with RI schedules. To this end, mice were first mildly food restricted (3 g/d) and trained on a FR1 lever pressing task until acquisition criteria were met (see Materials and Methods; Extended Data Fig. 2-1*A*) and subsequently split into three groups which followed RI30/RI60 schedules under different food restriction conditions (No Restriction, Mild Restriction, Strong Restriction; Fig. 2*A*). Animals had either unlimited access to food in their home cage (No Restriction), received 3 g of chow per day (Mild Restriction) or received only 2 g of chow per day (Strong Restriction; Extended Data Fig. 2-1*B*). For each group and each phase of training, we determined the mean response rate as well as the mean reinforcer delivery rate and computed the RR schedule at which the responses-per-reinforcer ratio would match that of the RI schedule given the observed response rates (defined as response rate/reinforcer delivery rate; Fig. 2*B*,*C*).

We then trained three groups on the identified RI-matched RR schedules for each food restriction condition (Fig. 2*D*) and compared the responses-per-reinforcer ratios during each phase of training to validate our strategy. During Phase 1, a two-way ANOVA revealed that there was a main effect of restriction group but not of task schedule (RI_NoRestriction: 2.74 ± 0.19, *N* = 10; RR_NoRestriction: 3.61 ± 0.06, *N* = 9; RI_MildRestriction: 4.08 ± 0.32, *N* = 14; RR_MildRestriction: 3.98 ± 0.03, *N* = 15; RI_StrongRestriction: 5.34 ± 0.36, *N* = 14; RR_StrongRestriction: 5.80 ± 0.05, *N* = 10; two-way ANOVA, effect of task: df = 1, *F* = 3.02, *p* = 0.087; effect of restriction: df = 2, *F* = 47.4, *p*= 1.70e-13). *Post hoc* comparisons confirmed there were no significant differences between each pair of restriction-matched RI-RR group (RI_NoRestriction-versus-RR_NoRestriction Tukey’s HSD *p*-adjusted = 0.22; RI_MildRestriction-versus-RR_mildRestriction Tukey’s HSD *p*-adjusted = 1.0; RI_StrongRestriction-versus-RR_StrongRestriction Tukey’s HSD *p*-adjusted = 0.76). Results during phase two were similar (RI_NoRestriction: 3.25 ± 0.25, *N* = 10; RR_NoRestriction: 4.18 ± 0.11, *N* = 9; RI_MildRestriction: 6.19 ± 0.49, *N* = 14; RR_MildRestriction: 6.06 ± 0.21, *N* = 15; RI_StrongRestriction: 9.33 ± 0.39, *N* = 14; RR_StrongRestriction: 9.98 ± 0.08, *N* = 10; two-way ANOVA, effect of task: df = 1, *F* = 2.23, *p* = 0.14; effect of restriction: df = 2, *F* = 145, *p* = 6.65e-25) and *post hoc* tests again confirmed no differences between each pair of restriction-matched RI-RR group (RI_NoRestriction-versus-RR_NoRestriction Tukey’s HSD *p*-adjusted = 0.50; RI_MildRestriction-versus-RR_mildRestriction Tukey’s HSD *p*-adjusted = 1.0; RI_StrongRestriction-versus-RR_StrongRestriction Tukey’s HSD *p*-adjusted = 0.75). Thus, our training strategy successfully produced three pairs of RI-RR groups with distinct levels of food restrictions and matched responses-per-reinforcer ratios, allowing us to compare the effects of restriction and task schedule on behavior and outcome devaluation.

### Food restriction increases response rates more effectively in mice following RR schedules than in mice following RI schedules

We first analyzed differences in performance (Fig. 3*A*) by testing how food restriction and task schedule influenced response rates by the end of training (Fig. 3*B*, RI_NoRestriction: 1.43 ± 0.19 responses/minute, *N* = 10; RR_NoRestriction: 1.66 ± 0.29 responses/minute, *N* = 9; RI_MildRestriction: 4.48 ± 0.67 responses/minute, *N* = 14; RR_MildRestriction: 7.15 ± 1.19 responses/minute, *N* = 15; RI_StrongRestriction: 9.17 ± 0.37 responses/minute, *N* = 14; RR_StrongRestriction: 16.3 ± 2.41 responses/minute, *N* = 10) and found that both restriction and task schedule had a main effect on response rate (two-way ANOVA, effect of task: df = 1, *F* = 15.2, *p* = 2.3e-4; effect of restriction: df = 2, *F* = 45.6, *p* = 3.6e-13). The interaction term was also significant (two-way ANOVA, interaction between task and restriction df = 2, *F* = 4.64, *p* = 0.013), suggesting restriction and task schedule interacted to influence operant behavior. These effects were similar when we analyzed reinforcer delivery rates (Extended Data Fig. 3-1*A*,*B*). To further test whether restriction and schedule combined to influence task acquisition, we repeated this analysis using the fold change in response rate between the last and first session of training after FR1 acquisition (Fig. 3*C*, RI_NoRestriction: 0.64 ± 0.12 responses/minute, *N* = 10; RR_NoRestriction: 0.53 ± 0.11 responses/minute, *N* = 9; RI_MildRestriction: 0.90 ± 0.11 responses/minute, *N* = 14; RR_MildRestriction: 1.25 ± 0.19 responses/minute, *N* = 15; RI_StrongRestriction: 1.45 ± 0.13 responses/minute, *N* = 14; RR_StrongRestriction: 3.19 ± 0.79 responses/minute, *N* = 10). Like final response rates, the fold change in response rates was influenced by the interaction between food restriction level and task schedule (two-way ANOVA, effect of task: df = 1, *F* = 8.29, *p* = 6.9e-3; effect of restriction: df = 2, *F* = 15.7, *p* = 5.0e-6, interaction: df = 2, *F* = 4.83, *p* = 0.011).

Next, we asked whether schedule and restriction differentially affected response variability. We analyzed the inter-response interval coefficients of variation (defined as SD/mean) across restriction groups and schedules and found that while RI animals respond with more regular intervals, stronger food restriction also promoted low variability (Extended Data Fig. 3-1*C*,*D*, RI_ NoRestriction: 1.03 ± 0.09, *N* = 10; RR_NoRestriction: 1.24 ± 0.06, *N* = 9; RI_MildRestriction: 0.59 ± 0.11, *N* = 14; RR_MildRestriction: 0.83 ± 0.10, *N* = 15; RI_StrongRestriction: 0.35 ± 0.04, *N* = 14; RR_StrongRestriction: 0.61 ± 0.0, *N* = 10; two-way ANOVA, effect of task: df = 1, *F* = 11.1, *p* = 1.4e-3). Unlike for response rate, there was clearly no interaction between schedule and restriction (effect of restriction: df = 2, *F* = 26.2, *p* = 4.3e-9, interaction: df = 2, *F* = 0.026, *p* = 0.97).

We draw three conclusions from these results. First, they show that when the responses-per-reinforcer ratios are matched, mice trained on RI schedules do not achieve a higher response rate than their counterparts trained on RR schedules under any circumstances. Second, both food restriction and task schedule independently influenced reinforcer delivery rate and response rate, with more restricted mice and mice on RR schedules achieving higher rates than less restricted mice and mice on RI schedules, respectively. Response rate variability was also independently influenced by each factor such that RI schedules and strong restriction promoted lower variability compared with RR schedules and mild restriction, respectively. Third, there was a clear combinatorial effect of restriction and task schedule: food restriction increased reinforcer delivery rates and response rates more effectively in mice following RR schedules than in mice following RI schedules, but they did not interact to affect response rate variability.

### The effects of extinction dominate the behavioral read-out during devaluation tests and are sensitive to food restriction but not to schedule of reinforcement

After completing the 7 d of RI or RR training, all mice underwent sequential outcome devaluation sessions. Animals were first prefed the reinforcer (devalued condition) or chow (control, valued condition) and subsequently tested in a 10-min extinction session in which responses have no programmed consequences. On the following day, each mouse underwent the same test with the prefeed it did not receive on the first day (for more details, see Materials and Methods). Thus, both sensory-specific satiety and degradation of the action-outcome relationship experienced during the extinction session have the potential to influence response rates. To establish the effectiveness of the devaluation procedure, a subset of mice further performed a preference test following each extinction session during which it had access to both the sucrose reinforcer and chow (Fig. 4*A*; Extended Data Fig. 4-1). Mice who had *ad libitum* access to food were not included in this analysis because they responded with rates too low to detect a decrease. The raw results of each session for each mouse are depicted in Figure 4*B* and a three-way ANOVA was performed on the devaluation index derived from these data (Fig. 4*C*, left, RI_MildRestriction_first: 0.52 ± 0.07, *N* = 4; RI_MildRestriction_second: 0.44 ± 0.05, *N* = 4; RR_MildRestriction_first: 0.48 ± 0.06, *N* = 5; RR_MildRestriction_second: 0.39 ± 0.04, *N* = 5; RI_StrongRestriction_first: 0.66 ± 0.03, *N* = 8; RI_StrongRestriction_second: 0.32 ± 0.04, *N* = 6; RR_StrongRestriction_first: 0.58 ± 0.04, *N* = 5; RR_StrongRestriction_second: 0.34 ± 0.10, *N* = 4) to identify the effects of task schedule, food restriction and session order on outcome devaluation. We found that only session order had a significant main effect on the devaluation index (three-way ANOVA, main effect of order df = 1, *F* = 32.1, *p* = 3.0e-6) while task schedule and restriction level had no effect (three-way ANOVA, main effect of schedule df = 1, *F* = 1.18, *p* = 0.28; main effect of restriction df = 1, *F* = 0.42, *p* = 0.52). To illustrate this result, we plotted the means for groups collapsed by task schedule, restriction, and session order (Fig. 4*C*, right). In addition, there was a significant interaction between restriction and session order (df = 1, *F* = 7.7, *p* = 9.1e-3). Taken together, these results show that when performing a devaluation test with sequential extinction sessions, the observed decrease in response rates largely reflects the effects of the degraded action-outcome relationship experienced by animals during the extinction sessions.

Because our analysis of devaluation indices revealed an interaction between session order and restriction in addition to the clear effect of order, we sought to determine how food restriction affected extinction across the two sessions (Fig. 4*D*). Similar to the devaluation index, we computed an extinction index and asked whether it was influenced by food restriction or task schedule (Fig. 4*E*, left, RI_MildRestriction: 0.46 ± 0.04, *N* = 8; RR_MildRestriction: 0.46 ± 0.04, *N* = 10; RI_StrongRestriction: 0.33 ± 0.03, *N* = 14; RR_StrongRestriction: 0.38 ± 0.05, *N* = 9). A two-way ANOVA revealed that only food restriction, but not schedule, influenced the magnitude of the decrease in response rate from the first extinction session to the second extinction session (two-way ANOVA, main effect of schedule df = 1, *F* = 0.54, *p* = 0.47; main effect of restriction df = 1, *F* = 7.1, *p* = 0.011; interaction: df = 1, *F* = 0.41, *p* = 0.53). To illustrate this result, we plotted the means for groups collapsed by task schedule and restriction (Fig. 4*E*, right). Thus, our results suggest that the observed decreases in response rates during sequential devaluation sessions largely reflect the effects of extinction and are sensitive to the food restriction state of the animal but not to the schedule used to reinforce behavior before the test.

### The effects of restriction are only apparent across days and not within an extinction session

Our results suggest that food restriction influences how rapidly animals decrease their response rates on experiencing a degradation of the action-outcome relationship underlying extinction sessions. To further explore this possibility, we plotted the response rates during the first extinction session across time (10 one-min bins; Fig. 5*A*). When we compared the response rates during the first minute (Fig. 5*B*, RI-MildRestriction: 6.25 ± 1.0, *N* = 8; RR-MildRestriction: 7.80 ± 1.55, *N* = 10; RI-StrongRestriction: 12.5 ± 2.1, *N* = 14; RR-StrongRestriction: 22.6 ± 4.3, *N* = 9), we found that both restriction and schedule had an effect on response rates (two-way ANOVA main effect of restriction group df = 1, *F* = 16.3, *p* = 2.6e-4; main effect of schedule df = 1, *F* = 5.99, *p* = 0.019), as would be predicted from the responses during training (Fig. 3). The interaction between schedule and restriction was not significant (df = 1, *F* = 2.7, *p* = 0.11), which would also be expected in the absence of reinforcement. However, when we repeated this analysis using an extinction index capturing the change in response rate from the first to second minute within the session (Fig. 5*C*, RI-MildRestriction: 0.38 ± 0.04, *N* = 8; RR-MildRestriction: 0.41 ± 0.07, *N* = 10; RI-StrongRestriction: 0.36 ± 0.04, *N* = 14; RR-StrongRestriction: 0.38 ± 0.04, *N* = 9), we found that neither restriction group nor schedule had a significant influence (two-way ANOVA main effect of restriction group df = 1, *F* = 0.21, *p* = 0.65; main effect of schedule df = 1, *F* = 0.26, *p* = 0.61; interaction df = 1, *F* = 0.0087, *p* = 0.93). This result was confirmed when we plotted the number of responses each animal produced before its inter-response interval surpassed 2 SDs above the mean of a rolling window of length five responses, a measure capturing the number of actions it took for mice to slow down their responses, as again neither restriction nor schedule showed a significant effect (Fig. 5*D*, RI-MildRestriction: 8.5 ± 1.31, *N* = 8; RR-MildRestriction: 8.8 ± 1.0, *N* = 10; RI-StrongRestriction: 9.0 ± 0.75, *N* = 14; RR-StrongRestriction: 9.11 ± 0.82, *N* = 9; two-way ANOVA main effect of restriction group df = 1, *F* = 0.18, *p* = 0.68; main effect of schedule df = 1, *F* = 0.041, *p* = 0.84; interaction df = 1, *F* = 0.0095, *p* = 0.92). These results show that while food restriction influenced extinction across days, it had no impact on the decrease in response rates observed during a single session.

## Discussion

Our results show that the levels of food restriction under which animals learn and operate affect their performance differently based on schedule of reinforcement. Indeed, food restriction increased response rates and reinforcer delivery rates more strongly in mice following RR schedules compared with mice following RI schedules. In parallel, animals were rendered more sensitive to extinction experienced during devaluation procedures by increasing levels of food restriction. Thus, task schedule fundamentally interacted with food restriction level to shape behavioral output during task acquisition, yet it showed little effect on behavior during extinction compared with food restriction level. Overall, our results show that schedule of reinforcement must be considered in combination with other factors that may influence motivation when attempting to identify the cognitive basis underlying operant behaviors.

Our results are consistent with previous studies showing that when reinforcer delivery rate is matched, animals following RR schedules generally achieve higher response rates than those following RI schedules – and this effect appears consistent across species (rats: Dawson and Dickinson, 1990; pigeons: Zuriff, 1970; Catania et al., 1977; Peele et al., 1984; humans: McDowell and Wixted, 1986; de Wit et al., 2018; Pérez et al., 2019). Consistent with the idea that RR schedules, by virtue of their strong action-outcome correlation (Ferster and Skinner, 1957; Dickinson, 1985; Pérez et al., 2019; although see Garr et al., 2020), allow for factors like motivation to influence response rates, we found that response rates were more sensitive to food restriction level in mice trained on RR schedules compared with RI schedules. However, whether the effects of food restriction and schedule of reinforcement interact differently across species is less clear. For example, previous work has shown that while training schedule has little effect on motivation in mice (Johnson et al., 2022), food restriction does impact measures of motivation in both rats (Dietze et al., 2016; Maliković et al., 2018) and mice (Mifune et al., 2020). The effects of schedule specific features, such as the preferential reinforcement of long inter-response times (IRTs) in RI schedules (Krame and Rilling, 1970; Kuch and Platt, 1976) or task parameters more generally (Follman et al., 2023), may also influence behavior in distinct species differently. By manipulating food restriction levels, our findings provide further support to the notion that the ability to control one’s own reinforcement rate explains the difference in response rates generated by RR and RI schedules in mice.

Several studies have explored the effects of changes in reinforcer value or in food restriction levels on performance (Sidman and Stebbins, 1954; Powell, 1969; Lowe et al., 1974; Blakely and Schlinger, 1988; Schlinger et al., 1990; Balleine, 1992; Parkes et al., 2017). However, these studies were largely focused on evaluating the effects of acute changes in value or in feeding schedule rather than understanding the systematic relationship between food restriction and reinforcement structure. Nevertheless, changes in reinforcer value have been shown to have a stronger effect on actions temporally close to the reinforcer delivery compared with those further away (Hollland and Rescorla, 1975; Dickinson and Balleine, 1995). Our findings support this idea because they suggest that tasks that promote a high response rate, either via food restriction or via richer schedules, are more likely to appear sensitive to value. This is also supported by the observation that leaner RI schedules are more likely to produce devaluation-insensitive behaviors (Garr et al., 2020) and that as long as animals continue to perform and remain in control of their own reinforcement rate, they remain goal-directed even for long periods of time (Colwill and Rescorla, 1985, 1988; Colwill and Triola, 2002; Corbit et al., 2012; Garr et al., 2021). We also found that stronger food restriction, together with RI schedules of reinforcement, promoted less variable response rates, however it is not clear whether these effects are related. Indeed, strong food restriction may increase task engagement and thus continued responding, while RI schedule may promote stable response rates by maintaining relatively high uncertainty as to whether a response will produce a reinforcer. Overall, our findings support the idea that schedule-dependent and schedule-independent factors interact to influence behavior and that studies must carefully match reinforcement rates, intrinsic reinforcer value and exposure to action-outcome contingencies when using response rates as a measure of operant behavior.

The results from our devaluation experiments reveal several insights. First, a limitation of our study is that we used chow as both regular food and as the control during devaluation procedures. Thus, we cannot completely rule out that prefeeding on sucrose versus chow did not influence the animals’ motivation and engagement differently. Nonetheless, our results do not support a scenario in which more strongly restricted animals experienced a larger decrease in motivation following prefeeding as all groups showed similar drops in their response rate within the first extinction session (Fig. 5). Instead, our results suggest that food restriction affected the ability of animals to remember the degraded action-outcome relationship experienced in extinction session 1 the following day (Fig. 4*D*,*E*). This effect supports a role for energy homeostasis in memory consolidation that has been observed in other learning contexts (Diano et al., 2006; Verma et al., 2016) but is rarely considered in operant conditioning. Second, we performed the devaluation procedures sequentially, without re-training between days. While this approach is commonly used (Parkes et al., 2017; Renteria et al., 2018; Hadjas et al., 2019), we show here that under these circumstances, the testing order dominates the observed decreases in response rate (Fig. 4*B*,*C*), not the valued versus devalued state of the animal, despite the confirmation that devaluation procedures selectively reduced the value of the prefed food (Extended Data Fig. 4-1). This result is a clear indication that counter-balancing the order in which reinforcer and control food are devalued is essential when attempting to determine sensitivity to value. Shorter probe tests and retraining between extinction sessions can also reduce the effects of extinction. While devaluation and extinction likely independently contribute to behavior, as revealed when training and extinction are performed in different contexts (Thrailkill and Bouton, 2015) or when multiple levers with distinct contingencies to reward are available (Dickinson et al., 1998), our results show that the interplay between these two factors must be carefully accounted for when interpreting devaluation procedures.

Finally, our results add to the growing evidence against a clear-cut relationship between schedule and cognitive basis of behavior, in which RR training produces goal-directed actions and RI training produces habitual actions (Colwill and Rescorla, 1990; Dezfouli and Balleine, 2012; de Wit et al., 2018; Garr and Delamater, 2019; Schreiner et al., 2020). While the two schedules produce inherently distinct features such as reinforcement uncertainty (de Russo et al., 2010), the temporal distribution of reinforcers and of responses (Adams, 1982; Doughty and Lattal, 2003; Urcelay and Jonkman, 2019), the correlation between action and outcome or the preferential reinforcement of long IRTs (Krame and Rilling, 1970; Kuch and Platt, 1976), that all likely contribute to differences in value sensitivity, external factors are at least equally important. Our data show that food restriction, together with schedule of reinforcement, affects how animals learn and support evidence that external factors such as reinforcer value, on-board drug or stimulus saliency (Vandaele et al., 2017), both contribute to, and can fundamentally control, the underlying cognitive basis of behavior.

## Synthesis

Reviewing Editor: Christie Fowler, University of California Irvine Decisions are customarily a result of the Reviewing Editor and the peer reviewers coming together and discussing their recommendations until a consensus is reached. When revisions are invited, a fact-based synthesis statement explaining their decision and outlining what is needed to prepare a revision will be listed below. The following reviewer(s) agreed to reveal their identity: Christopher Olsen.

The authors have thoroughly addressed both reviewers’ concerns. The manuscript reports a complex relationship between reinforcement schedule and the impact of food restriction level. The interaction between schedule and food restriction was primarily present during task acquisition, while food restriction level was the primary factor associated with differences in responding during devaluation. Furthermore, food restriction level affected between-session extinction rate more than within-session response rates during devaluation. We appreciate the new data and analyses. With these new data it is clear that test order and extinction were, indeed, important contributors to the results. It was also appreciated how the authors have now reframed the interpretation in this light. It was further felt that the transparency of the data will be useful to scientists looking to use these methods. To that end, the reviewers felt it would be of benefit to address the following points:

1. In the discussion, please provide some suggestions to scientists who are using food deprivation levels, schedules of reinforcement, and devaluation. Clearly, test order and extinction have to be accounted for. But, also, the authors have likely found several other important factors: counterbalancing trained outcome, use outcomes matched for value and exposure, shorter probe tests and retraining in between tests will help avoid the influence of extinction.

2. Avoid the use of “degraded action-outcome contingency/degradation of the action-outcome contingency” to refer to extinction. Contingency degradation is a term that has been specifically used to refer to a scenario in which the action still earns the reward but the reward is also non-contingently delivered with the same probability. Using it to describe extinction may, therefore, be confusing for some communities.

3. On line 16, please edit the sentence to clarify that food restriction accelerated the devaluation-associated decrease in response rates.
