# Supplementary figures and images for: Food Restriction Level and Reinforcement Schedule Differentially Influence Behavior during Acquisition and Devaluation Procedures in Mice
Source: eNeuro. 2023 Sep 26;10(9):ENEURO.0063-23.2023. doi: 10.1523/ENEURO.0063-23.2023 (PMC10537440; doi:10.1523/ENEURO.0063-23.2023)

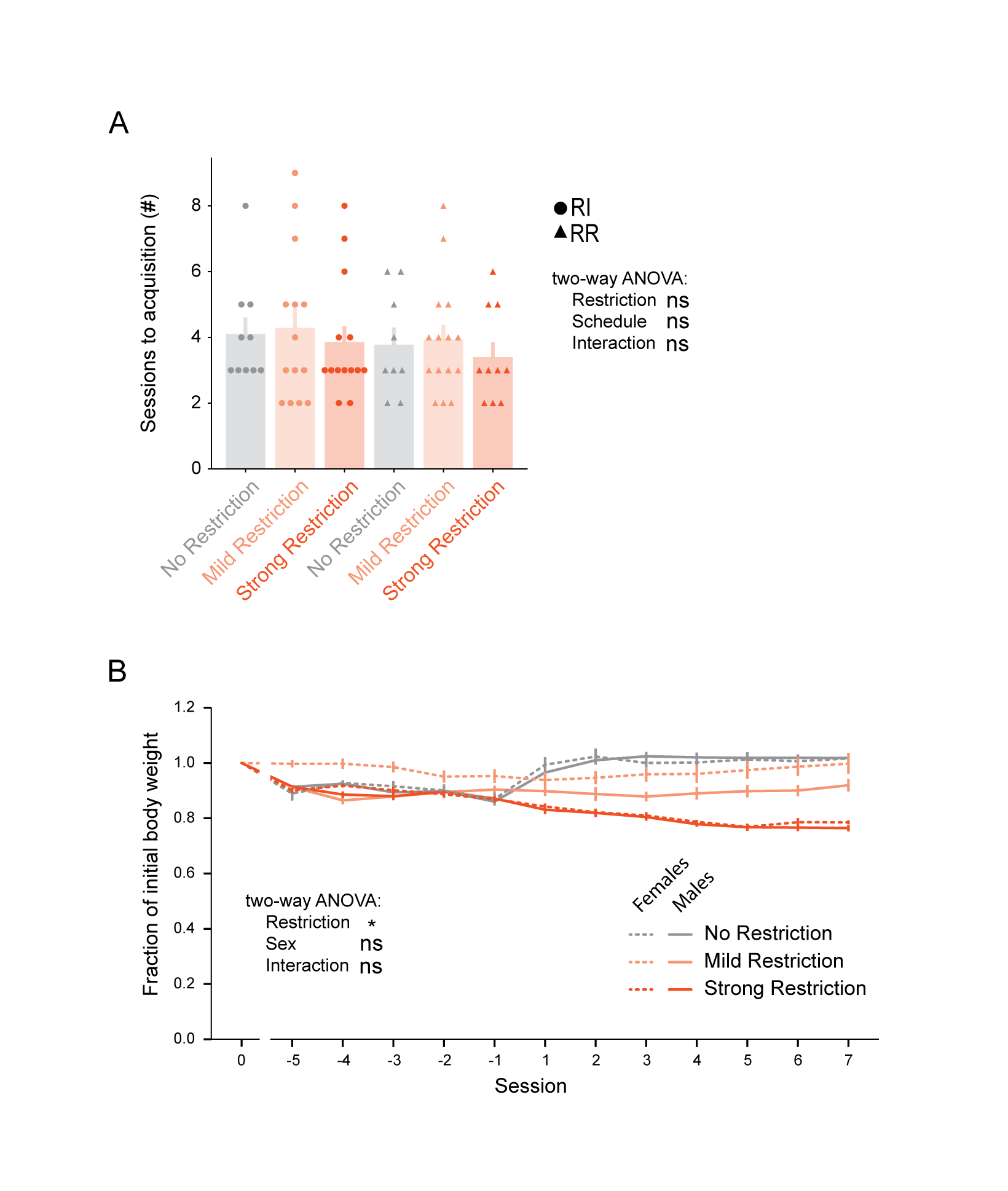

Supplement: Extended Data Figure 2-1 — No difference in time to acquisition across restriction/schedule and in the efficacy of food restriction across sex. A, Graph showing the time to acquisition across training schedules and restriction groups [RI-NoRestriction: 4.1 ± 0.5 sessions (N = 10), RI-MildRestriction: 4.29 ± 0.62 sessions (N = 14), RI-StrongRestriction: 3.86 ± 0.49 sessions (N = 14), RR-NoRestriction: 3.78 ± 0.52 sessions (N = 9), RR-MildRestriction: 3.93 ± 0.45 sessions (N = 15), RR-StrongRestriction: 3.4 ± 0.45 sessions (N = 10)]. A two-way ANOVA revealed no effects of restriction group (two-way ANOVA F = 0.45, df = 2, p = 0.64) and no effect of task schedule (two-way ANOVA F = 0.77, df = 1, p = 0.38). These results are expected as we assigned mice to groups post FR1 acquisition to avoid any differences. B, Graph showing the effects of food restriction across time as the fraction of initial body weight [data on session 7: Females/NoRestriction: 1.02 ± 0.02 (N = 9), Females/MildRestriction: 0.99 ± 0.03 (N = 10), Females/StrongRestriction: 0.79 ± 0.01 (N = 12), Males/NoRestriction: 1.02 ± 0.01 (N = 10), Males/MildRestriction: 0.92 ± 0.02 (N = 10), Males/StrongRestriction: 0.76 ± 0.01 (N = 12)]. A two-way ANOVA on the last day (session 7) revealed a significant effect of restriction group (two-way ANOVA F = 90.1, df = 2, p = 2.3e-18) but no effect of sex (two-way ANOVA F = 3.48, df = 1, p = 6.7e-2). Download Figure 2-1, TIF file. [file enu-eN-NWR-0063-23-s02.tif]

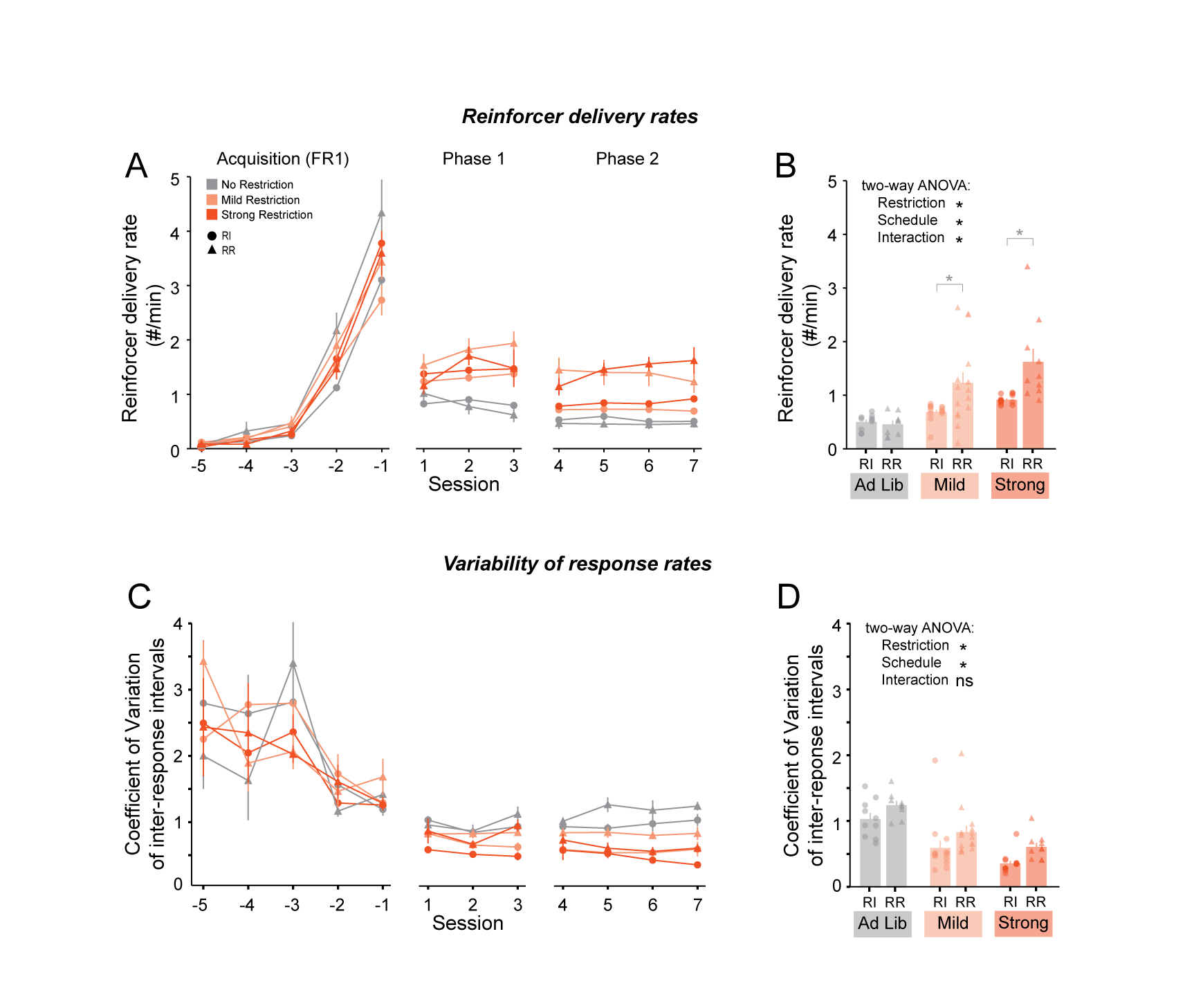

Supplement: Extended Data Figure 3-1 — Food restriction and schedule influenced reinforcer delivery rates and response rate variability. A, Mean reinforcer delivery rates across training, split by restriction and schedule. B, Mean reinforcer delivery rates on the last day of training summarizing the data used to perform a two-way ANOVA. C, Mean coefficient of variation of the inter-response interval across training, split by restriction and schedule. D, Mean coefficient of variation on the last day of training summarizing data used to perform a two-way ANOVA. RI-noRestriction N = 10 mice, RR-NoRestriction N = 9 mice, RI-MildRestriction N = 14 mice, RR-MildRestriction N = 15 mice, RI-strongRestriction N = 14 mice, RR-strongRestriction N = 10 mice; black * indicates two-way ANOVA p < 0.05 (B, D); grey * indicates p < 0.05 post hoc Tukey’s HSD test for matched RR-RI pairs (B, D). ANOVA results and post hoc Tukey’s HSD tests are reported in the statistical table. Data are shown as mean ± SEM. Individual data points are shown separated by sex within each group (Females: left, Males: right). Download Figure 3-1, TIF file. [file enu-eN-NWR-0063-23-s03.tif]

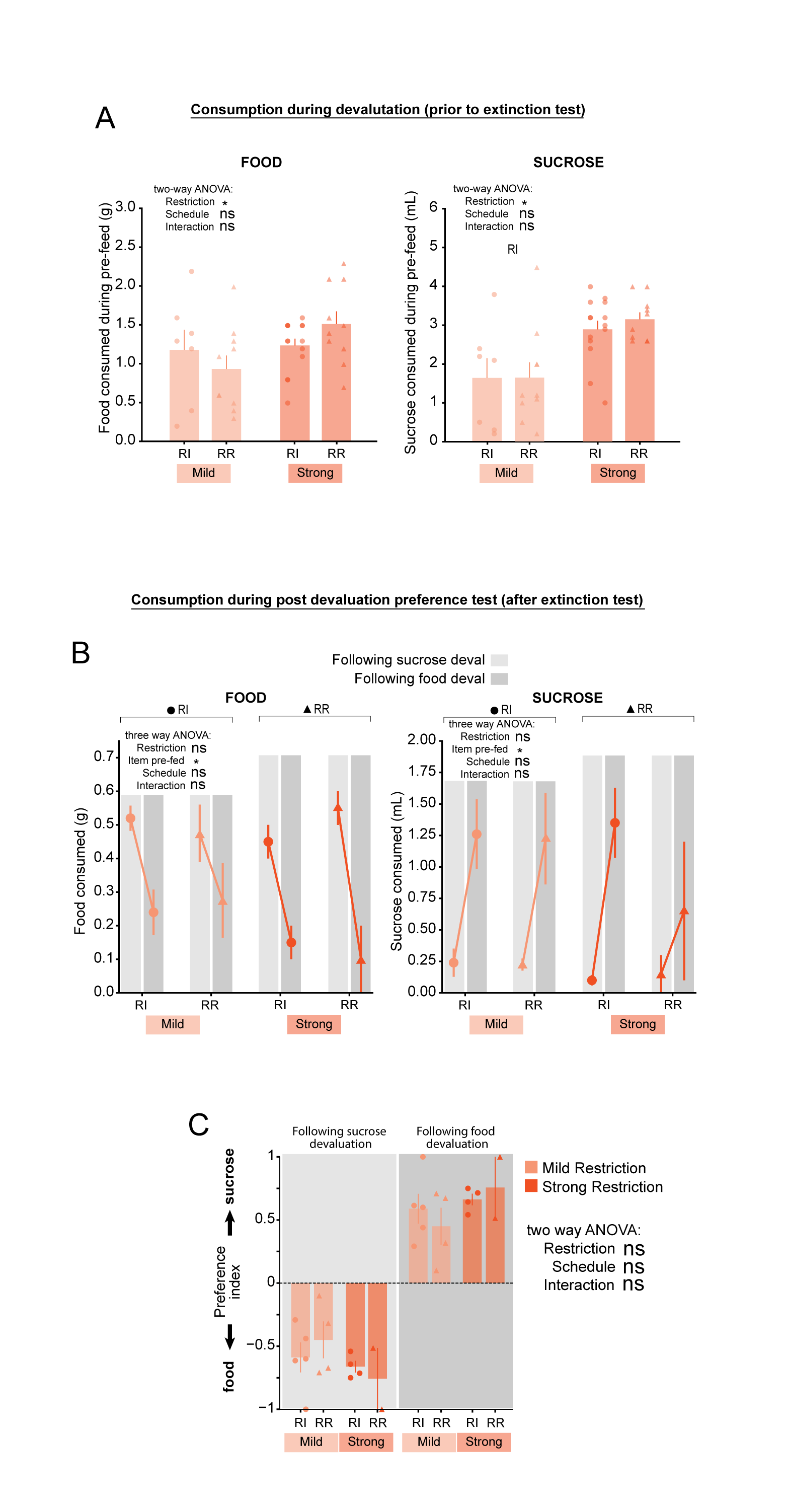

Supplement: Extended Data Figure 4-1 — Prefeeding selectively and effectively reduced mice’s preference for the prefed food. A, Graphs showing how much food [left; RI-MildRestriction: 1.08 ± 0.24 g (N = 8), RR-MildRestriction: 0.94 ± 0.17 g (N = 10), RI-StrongRestriction: 1.25 ± 0.09 g (N = 14), RR-StrongRestriction: 1.52 ± 0.16 g (N = 10); two-way ANOVA main effect of restriction group df = 1, F = 4.35, p = 0.044; main effect of schedule df = 1, F = 0.15, p = 0.70; interaction df = 1, F = 2.39, p = 0.13) or sucrose (right; RI-MildRestriction: 1.62 ± 0.44 g (N = 8), RR-MildRestriction: 1.65 ± 0.40 g (N = 10), RI-StrongRestriction: 2.9 ± 0.22 g (N = 14), RR-StrongRestriction: 3.16 ± 0.18 g (N = 10); two-way ANOVA main effect of restriction group df = 1, F = 18.6, p = 1.15e-4; main effect of schedule df = 1, F = 0.24, p = 063; interaction df = 1, F = 0.15, p = 0.70] mice consumed during the 1 h prefeeding session preceding the extinction test. B, Graphs showing consumption during the postdevaluation preference test, during which mice have free access to both food and sucrose for 10 min, for food [left; RI-MildRestriction_postSucDeval: 0.52 ± 0.04 g (N = 5), RI-MildRestriction_postFoodDeval: 0.24 ± 0.07 g (N = 5), RR-MildRestriction_postSucDeval: 0.48 ± 0.09 g (N = 4), RR-MildRestriction_postFoodDeval: 0.27 ± 0.11 g (N = 4), RI-StrongRestriction_postSucDeval: 0.45 ± 0.05 g (N = 4), RI-StrongRestriction_postFoodDeval: 0.15 ± 0.05 g (N = 4), RR-StrongRestriction_postSucDeval: 0.55 ± 0.05 g (N = 2), RR-StrongRestriction_postFoodDeval: 0.10 ± 0.10 g (N = 2); three-way ANOVA main effect of restriction group df = 1, F = 1.68, p = 0.21; main effect of schedule df = 1, F = 0.014, p = 0.91; main effect of prefeed df = 1, F = 30.8, p = 1.4e-5; interactions all showed p > 0.05) and for sucrose (right; RI-MildRestriction_postSucDeval: 0.24 ± 0.11 g (N = 5), RI-MildRestriction_postFoodDeval: 1.26 ± 0.28 g (N = 5), RR-MildRestriction_postSucDeval: 0.22 ± 0.05 g (N = 4), RR-MildRestriction_postFoodDeval: 1.22 ± 0.36 g (N [file enu-eN-NWR-0063-23-s04.tif]
